# Deep learning network for integrated coil inhomogeneity correction and brain extraction of mixed MRI data

**DOI:** 10.1038/s41598-022-12587-6

**Published:** 2022-05-20

**Authors:** Kai-Hsiang Chuang, Pei-Huan Wu, Zengmin Li, Kang-Hsing Fan, Jun-Cheng Weng

**Affiliations:** 1 Queensland Brain Institute and Centre for Advanced Imaging, University of Queensland, Brisbane, Australia; 2 Department of Medical Imaging and Radiological Sciences, and Graduate Institute of Artificial Intelligence, Chang Gung University, No. 259, Wenhua 1st Rd., Guishan Dist., Taoyuan, 33302 Taiwan; 3 Department of Radiation Oncology, Chang Gung Memorial Hospital at Linkou, Taoyuan, Taiwan; 4 Medical Imaging Research Center, Institute for Radiological Research, Chang Gung University and Chang Gung Memorial Hospital at Linkou, Taoyuan, Taiwan; 5 Department of Psychiatry, Chang Gung Memorial Hospital, Chiayi, Taiwan

**Keywords:** Brain imaging, Functional magnetic resonance imaging, Magnetic resonance imaging, Image processing, Machine learning

## Abstract

Magnetic Resonance Imaging (MRI) has been widely used to acquire structural and functional information about the brain. In a group- or voxel-wise analysis, it is essential to correct the bias field of the radiofrequency coil and to extract the brain for accurate registration to the brain template. Although automatic methods have been developed, manual editing is still required, particularly for echo-planar imaging (EPI) due to its lower spatial resolution and larger geometric distortion. The needs of user interventions slow down data processing and lead to variable results between operators. Deep learning networks have been successfully used for automatic postprocessing. However, most networks are only designed for a specific processing and/or single image contrast (e.g., spin-echo or gradient-echo). This limitation markedly restricts the application and generalization of deep learning tools. To address these limitations, we developed a deep learning network based on the generative adversarial net (GAN) to automatically correct coil inhomogeneity and extract the brain from both spin- and gradient-echo EPI without user intervention. Using various quantitative indices, we show that this method achieved high similarity to the reference target and performed consistently across datasets acquired from rodents. These results highlight the potential of deep networks to integrate different postprocessing methods and adapt to different image contrasts. The use of the same network to process multimodality data would be a critical step toward a fully automatic postprocessing pipeline that could facilitate the analysis of large datasets with high consistency.

## Introduction

Advanced MRI techniques, such as diffusion tensor imaging (DTI), arterial spin labeling (ASL) and functional MRI (fMRI), have become essential tools to describe the structural and functional organization of the human brain to diagnose disorders non-invasively in humans and to resolve neural mechanisms and treatment targets using rodent or other animal models. With the construction of brain atlases and templates, information-rich multimodality data obtained from different subjects at different times can be co-registered to a common reference space for voxel-wise and regional analyses to describe the effects of aging, disorders and therapeutics. To achieve accurate co-registration, images must be corrected for head motion, geometric distortion, and coil B1 field inhomogeneity. Then, the brain is extracted by removing signals from the scalp and other tissues before linear/nonlinear transformation can be applied to match the individual brain images to the template. Because inferior inhomogeneity correction and brain extraction can result in dislocated or biased findings^1^, methods to improve the intensity uniformity and skull stripping are essential.

To allow automatic data processing, various methods have been developed for coil inhomogeneity correction, such as N4^2^ and brain extraction, such as BET^3^, MONSTR^4^, 3D PCNN^5^, RATS^6^, and SHERM^7^. However, manual adjustments and editing are still required to obtain optimal results^8^. The need for user intervention results in slow data processing and variable quality from different users, which affects results. Also, these algorithms are typically developed for structural MRI. Performance is degraded when applying to echo-planar imaging (EPI), which is widely used for DTI, ASL and fMRI, due to different contrasts (e.g., T2/T2*-weighted versus T1-weighted), spatial resolutions (e.g., 2–3 mm versus 1 mm) and geometric distortions. Because studies have suggested that direct registering EPI to the brain template would be more accurate^9^, methods that can perform well on EPI are required. In addition, most processing methods have been developed for humans and are typically difficult to apply to animal data due to the different anatomy and resolutions involved.

Deep learning neural networks have become one of the most popular techniques of image processing in recent years^10^. The development of deep learning was previously constrained by computing power. With the advance of general-purpose computing on graphics processing units^11^, an algorithm with a complex deep network topology of several hundred million trainable parameters can be completed within a few days, which allows the application of deep learning networks to various challenging biomedical image processing issues (for review, see^12^), including brain extraction and segmentation^13^, Nyquist ghost removal and motion correction^14–16^. For example, variants of 3D convolutional networks were initially successfully applied for brain extraction from single contrast T_1_-weighted MPRAGE data^17–19^ and expanded to multiple contrast modalities or even pathological brains using large multicenter multicontrast training data^20^. Yoganada et al. combined DenseNet and U-Net to segment gray matter/white matter/CSF from brain MR images^13^. They used a 3D framework with many trainable parameters to achieve high accuracy, but the kernel size and filters of the convolution layer were constrained to allow the topology to converge. Similar attempts have been explored recently for the brain extraction of non-human primates^21^ and rodents^22^. Conventional intensity correction methods are still required for preprocessing before deep learning-based brain extraction. A deep learning network has also been developed to correct coil inhomogeneity^23^. However, this method was developed for a rather uniform head or body coil; its performance for images acquired by highly inhomogeneous surfaces or array coils is thus unclear. Overall, current deep-learning-based methods have several limitations. First, they are primarily designed for structural MRI and are thus difficult to apply to EPI data, which suffer from inferior image quality. Second, although skull stripping networks could process multicontrast structural MRI, methods for EPI data are still optimized for a specific contrast and thus difficult to apply to spin-echo (T_2_-weighted) and gradient-echo (T_2_*-weighted) EPI, which are commonly used for DTI and fMRI, respectively. Third, these methods are all designed for a single postprocessing procedure. Therefore, the data processing pipeline would still be limited by user intervention being required in the remaining processing.

In this study, we address these issues of deep learning networks designed to overcome two limiting steps of the brain MRI processing pipeline: coil inhomogeneity correction and brain extraction. We solved these issues by using generative adversarial nets (GANs)^24,25^ with expansion of the 2D GAN model to 3D. Because brain extraction requires coil inhomogeneity correction, we trained GANs to combine these two separate processing together. To manage different image contrasts, particularly spin-echo and gradient-echo EPI, GANs were trained using either a single contrast or multiple contrasts. Results demonstrate high accuracy and consistency compared to manually adjusted automatic methods like N4 and PCNN.

## Results

### Model training

**Figure 1:**
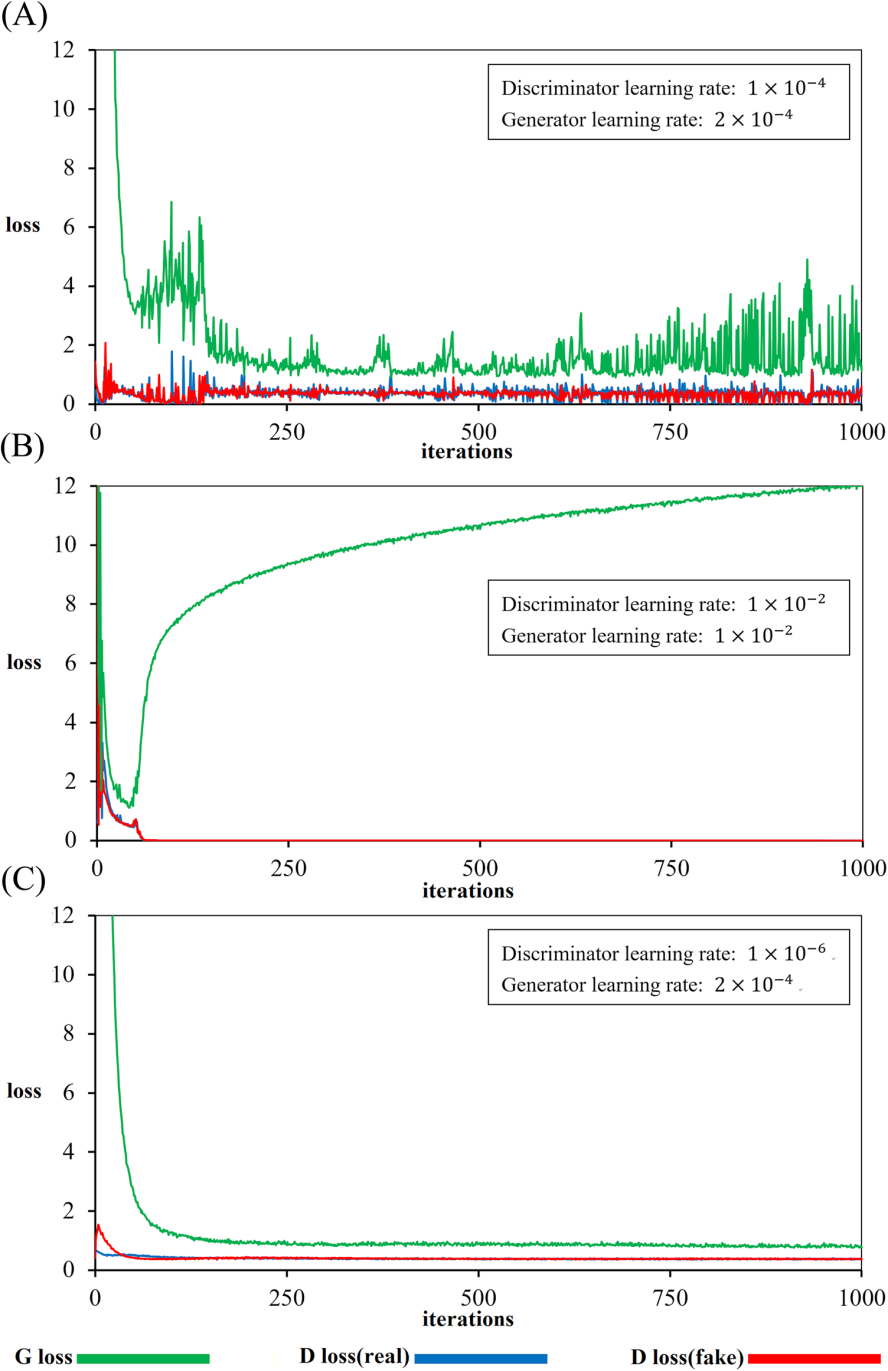
Effects of the learning rate on the training loss over iterations. (**A**) When the losses of the generator and discriminator strongly interact with each other, the outcome describes vibrations. (**B**) When the loss of the generator diverges, the model breakdowns, and the prediction from the model worsens. (**C**) With a proper learning rate, the loss smooth converges with stable results. The green line is the $\lambda {\mathcal{L}}_{L1}\left(G\right)+{\mathbb{E}}_{c}$ loss. The red and blue lines are cGAN losses formulated as ${\mathbb{E}}_{c,x}$ and ${\mathbb{E}}_{c}$, respectively. The curve is for the GE-EPI-only model.

In the GANs, the generator and discriminator compete with each other in a zero-sum game. Model training is similar to a seesaw battle: if one side is too strong, the loss function tends to oscillate strongly (Fig. 1A). If the vibration exceeds a certain critical point, the system has a high probability of breakdown, as shown in Fig. 1B. When that happens, the quality of the pseudoimage generated will worsen with more iterations. To prevent the model from oscillating, we use a small learning rate for the generator and discriminator ($2\times {10}^{-4}$ and $1\times {10}^{-6}$, respectively) in all the experiments. The Adam optimizer was used in both the discriminator and generator^26^. This process resulted in a stable trend, as shown in Fig. 1C.

Three types of GAN models were trained by gradient-echo EPI (GE-EPI) only, spin-echo EPI (SE-EPI) only and the mixed dataset. Supplementary Fig. 1 shows example results during training over 5 selected iterations using the GE-EPI-only dataset. Starting from a noisy image, the predicted image became increasingly similar to the correct target image when the GAN was trained with more iterations. With > 2000 iterations, the rough details of the brain were shown. With > 10,000 iterations, the predicted image became similar to the target. The histograms also became increasingly more similar to those of the target images, indicating the method’s effectiveness at removing the bias field of the surface coil. The models trained by the SE-EPI-only (Supplementary Fig. 2) or mixed (Supplementary Figs. 3 and 4) datasets showed similar converging trends. In particular, the model could remove the external tissue and the reference phantom over the rat’s head. The similarity indices of the training data also show progressive improvements with iterations (Supplementary Fig. 5).

### Choosing the best model

**Figure 2:**
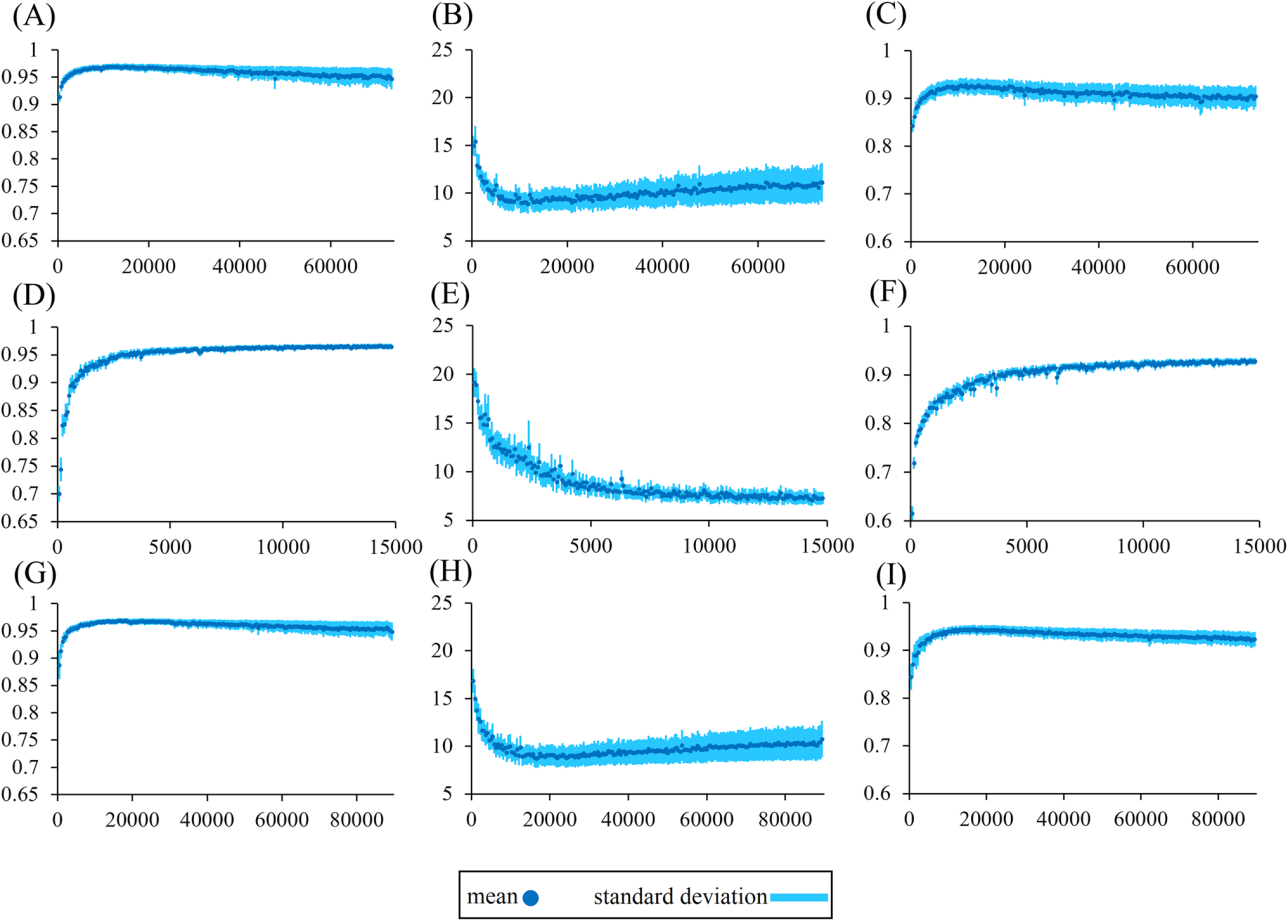
Similarity indices of test data over iterations. The CAD (first column), L2 norm (second column) and MSSIM (third column) of outputs from the GE-EPI-only (**A**–**C**), SE-EPI-only (**D**–**F**) and mixed (**G**–**I**) dataset-trained models. The mean and standard deviation are shown. The unit of the x-axis is the number of iterations.

With more iterations, the images generated by GANs became increasingly similar to the targets, which made it difficult to identify the best model. Because there is no good standard to determine how many iterations are required to train a model, we used a strategy that chose the model that could generate the best validation results. We calculated the similarity indices of the test data on all the models trained at each iteration and selected the one producing the best comprehensive performance as the final model. Figure 2 shows the similarity indices of the testing data for models over iterations. Then, we chose the model with 11,744 iterations for mouse-only, 14,356 iterations for rat-only, 16,539 iterations for mix dataset because they had the best mean cosine angle distance (CAD), Euclidean distance (L2 norm) and mean structural similarity (MSSIM). More specifically, we chose the model that had the highest mean MSSIM score in each experiment because the MSSIM was more consistent with visual inspection.

### Testing using data from the same modality

**Figure 3:**
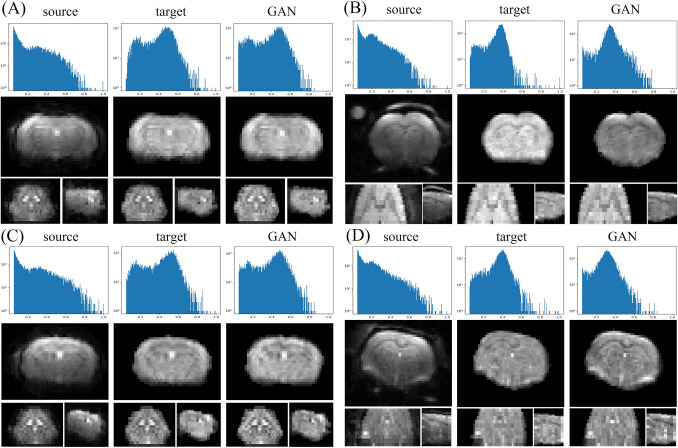
Example results of 3 experiments. From left to right are the histograms and tri-planar views of the source, target and GAN output images from the (**A**) GE-EPI-only model, (**B**) SE-EPI-only model, and (**C**, **D**) mixed model. (**A**) and (**C**) show the GE-EPI images, and (**B**) and (**D**) are the SE-EPI images.

Figure 3 shows representative results after applying GE-EPI or SE-EPI test data to the models trained by GE-EPI-only, SE-EPI-only and mixed datasets. Compared to the target image, the outputs of GANs show preserved tissue contrast but improved uniformity, with histograms similar to those of the target images. The GANs could even perform better than the target image. In Fig. 3B, the target image showed an abrupt intensity drop at the bottom of the brain, while the GAN output uniformly covered the entire brain. The target image in Fig. 3D appeared to be overcorrected for coil inhomogeneity so that the contrast between the gray and white matter was low. Conversely, the GAN output a uniform image with preserved tissue contrast. Interestingly, even when the SE-EPI and GE-EPI data have different image quality and features (e.g., external tissue intensity and phantom object), the performance of the mixed modality-trained model is comparable to that of the single-modality model. This result indicates that the *3D pix2pix* network could learn distinct features of the mixed dataset.

The performance of coil inhomogeneity correction was evaluated by comparing the similarity of intensity distribution, quantified using CAD, L2 norm and MSSIM (Table 1). The quality of brain extraction was quantified using the Dice index (Table 2). Overall, the performance of the GAN models was consistently high. Even though the training data exhibited a large deviation in the MSSIM for the GE-EPI only and mixed datasets due to the particular intensity distribution of certain subjects (see “Discussion” below), the performance of the testing data was consistent across subjects with a small standard deviation. The performance of the mixed-modality model was also comparable to that of the single-modality model.

**Table 1:** Similarity indices of test data for each corresponding model.

Model	Sample	Index	Mean	SD	Max	Min
GE-EPI only	Training (scan = 367)	CAD	0.994	0.003	0.996	0.968
		L2 norm	4.609	1.116	10.064	3.089
		MSSIM	0.968	0.074	0.989	0.368
	Test (scan = 36)	CAD	0.969	0.011	0.992	0.950
		L2 norm	8.872	2.076	12.106	4.249
		MSSIM	0.928	0.031	0.980	0.843
SE-EPI only	Training (scan = 74)	CAD	0.996	0.003	0.998	0.982
		L2 norm	3.070	1.274	9.060	1.595
		MSSIM	0.985	0.011	0.994	0.922
	Test (scan = 13)	CAD	0.966	0.009	0.978	0.949
		L2 norm	7.094	1.204	9.406	5.729
		MSSIM	0.930	0.012	0.947	0.908
Mix	Training (scan = 447)	CAD	0.994	0.004	0.997	0.966
		L2 norm	4.243	1.141	10.785	2.380
		MSSIM	0.977	0.062	0.994	0.379
	Test scan = 43)	CAD	0.969	0.010	0.991	0.948
		L2 norm	8.696	1.977	12.329	4.761
		MSSIM	0.944	0.019	0.984	0.895

*SD* standard deviation.

**Table 2:** Dice index of GANs and N4 + PCNN outputs.

Model	Sample (scan)	Method	Mean	SD	Max	Min
GE-EPI only	36 (GE-EPI)	N4 + PCNN vs target	0.917	0.032	0.961	0.834
		GANs vs target	0.927	0.026	0.968	0.857
SE-EPI only	13 (SE-EPI)	N4 + PCNN vs target	0.958	0.024	0.982	0.885
		GANs vs target	0.954	0.009	0.969	0.940
Mix	43 (mix)	N4 + PCNN vs target	0.926	0.036	0.982	0.834
		GANs vs target	0.931	0.025	0.971	0.874
	33 (GE-EPI)	N4 + PCNN vs target	0.915	0.032	0.961	0.834
		GANs vs target	0.923	0.024	0.964	0.874
	10 (SE-EPI)	N4 + PCNN vs target	0.964	0.010	0.982	0.948
		GANs vs target	0.956	0.008	0.971	0.943

### Testing using multimodality data

**Figure 4:**
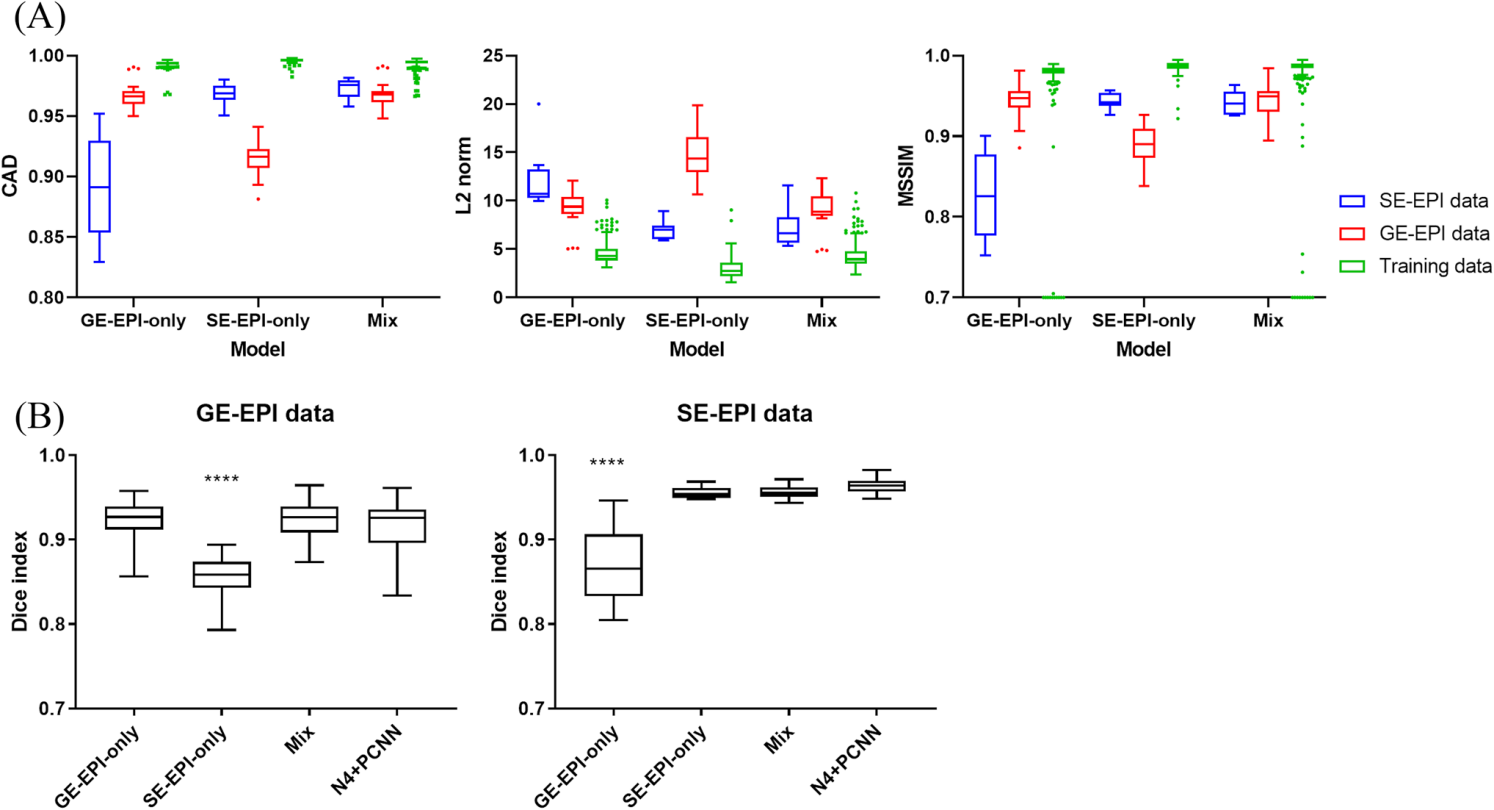
Performance comparison using mixed testing data. Box plots of (**A**) CAD, L2 norm and MSSIM, and (**B**) Dice index. ****p < 0.0001.

To evaluate how well the three models could manage data that were either familiar (same type of EPI as the training data) or unfamiliar (e.g., GE-EPI for SE-EPI-only trained), we applied the testing data for the mixed group to all three models and compared their performances (Fig. 4A). As expected, the test data of the same modality had high similarity indices, while the performance degraded markedly with unfamiliar data types. The performance on the test data was marginally inferior to that of the training data, which had similarity indices that were closer to ideal. Interestingly, the model trained by mixed data outperformed the model trained by the familiar but single modality data. For the CAD, the mixed model performed (0.973 ± 0.003; mean ± SEM) much better than the SE-EPI trained model (0.969 ± 0.003, p < 0.001) for the SE-EPI test data. For the GE-EPI test data, the mixed model performed (0.968 ± 0.002) marginally better than the GE-EPI trained model (0.966 ± 0.002, p < 0.05). Similar trends were also shown in the L2 norm and MSSIM. Therefore, training with more diverse data enhanced the capability of the network.

Using the combination of two popular automatic methods (N4 and PCNN) as a benchmark, we compared the Dice indices of the GAN outputs of the test data (Fig. 4B). To achieve the best results of the conventional methods, the parameters of N4 and PCNN were optimized for each individual. For the SE-EPI data, the Dice indices of models trained by the SE-EPI-only (0.955 ± 0.008) or mixed (0.956 ± 0.008) datasets were comparable to those of the N4 + PCNN (0.964 ± 0.010) but not the model trained by the GE-EPI-only data (0.871 ± 0.044; p < 0.0001). Similarly, the GE-EPI-only (0.923 ± 0.024) and mixed (0.923 ± 0.024) dataset trained models performed as well as the N4 + PCNN (0.915 ± 0.032), except for the SE-EPI-only trained model (0.858 ± 0.024; p < 0.0001). In addition, the mixed-trained model generally exhibited smaller variations than N4 + PCNN, indicating more consistent performance.

## Discussion

This study addressed two issues of deep-learning-based brain extraction: dependency on coil inhomogeneity and inflexibility for multimodality data. We used a deep learning network (3D pix2pix) to automate two critical and labor-intensive steps—coil inhomogeneity correction and brain extraction—in the brain EPI postprocessing pipeline. Results shows that different types of MRI postprocessing can be combined into one network, which can streamline the data processing pipeline and can reduce operator-dependent variations and bias in different processes. This network model can manage both spin- and gradient-echo EPI that are commonly used in DTI, ASL and fMRI. The capability of processing these major data types could facilitate the data analysis of the most commonly used advanced neuroimaging data. The model performed as well as existing automatic methods (N4 and PCNN) that were individually adjusted and optimized. Particularly, the results of the proposed method are more consistent than those from automatic methods. Although the proposed method was demonstrated using rodent data in this proof-of-concept study, similar network models could be built and trained using human data to streamline the workflow of clinical MRI data processing.

### Criteria for stopping the training of a model

There is no common standard about how many iterations are required to train model sufficiently. The original GAN paper mentioned that a global optimum solution exists for an ideal model such that the model iterated countless times will converge to the global optimum. In that case, fake data from the generator infinitely approach real data, and the discriminator can no longer distinguish real and fake data. Because there is no ideal model in reality, the proposed models may reach a local optimum solution^27,28^ or undergo mode collapse in training^29^. When we inspected the similarity indices calculated using the training dataset itself, the performance increased monotonically, and thus, the optimal performance would be achieved with infinite iterations. Therefore, we chose to use test data to select the best model. However, this approach suffers from the fact that the best model may be different for different test datasets. To overcome this issue, a more diverse testing dataset from different MRI scanners may be used to evaluate the overall performance across datasets from different sites.

### Performance of coil inhomogeneous correction

By visual inspection of the pseudoimages and their histograms generated by GANs, results were found to be similar to those of the target images, indicating good performance of the GANs in learning the corrected intensity distribution. To quantify the difference in the intensity distribution, we compared the CAD, L2 norm and MSSIM between the pseudo and target images (Table 1). The high similarity of the training data indicated that the model learned the importance features and relationships of the source and target images. Comparing the three datasets, the GE-EPI-only model had better performance than SE-EPI-only, which was likely due to the larger GE-EPI training dataset; thus, the model could learn more features for generating pseudoimages. Also, the image complexity of the SE-EPI data is much higher than that of the GE-EPI because the scalp and muscle signals are stronger in the SE-EPI in addition to the presence of an external phantom. Although the similarity indices of the SE-EPI-only model were inferior, the quality of the pseudoimage remained high, and only a few differences at the edge of the brain tissue could be identified. For the same reason, the similarity indices of the GE-EPI-only model were marginally worse than those of the mixed-trained model. Overall, there are many more distinct features in the mixed modality dataset that must be learned than in single-modality datasets. Under the same topology and hyperparameters, the mixed-trained model could learn complex features and achieved high similarity indices.

### Performance of brain extraction

Using the Dice index, the overlap between the ideal and automatically extracted brain masks was evaluated. Overall, the GANs achieved comparable accuracy compared to those of the two existing methods (N4 and PCNN) together. The extraction results of SE-EPI were better than those of GE-EPI, likely due to better image contrast and less blurring.

The two tasks (inhomogeneity correction and brain extraction) can potentially be processed by two network models separately. For example, GAN is used for the first step, and U-Net is used for the second step. When using two models in two stages to map two data distributions, it could suffer from error propagation. The first model may contribute some error and lead to more error in the prediction of the second model. The first model will also require more time to fine-tune the hyperparameters and training, as well as additional storage space for saving weights. Here we demonstrated that GANs can successfully combine these two processing in one network.

### Testing GANs using multimodality data

To evaluate how a trained model performs with data that are completely different from the training data, we tested the model trained by a single modality (i.e., GE-EPI-only and SE-EPI-only) dataset with the test data of the mixed model. The outcome was, as expected, not ideal when the data type was unfamiliar. The model trained by the SE-EPI-only data performed better than the model trained by GE-EPI-only, likely due to more features in the SE-EPI data that allow the model to process GE-EPI data but not conversely.

### Comparison to other network models

Deep learning has been applied to extract or correct different types of structural information, such as brain tumors, gray/white matter tissue segmentation, skull stripping or EPI distortion correction. Although these methods all belongs to image segmentation, each has its own special focus and challenge. Brain tissue segmentation focuses on classifying voxels of different intensity distributions with less consideration of morphology. Brain extraction focuses on identifying the boundary of the brain while ignoring intensity distributions of different tissue types. Recently, a few studies have used a popular deep learning network, U-net, for automatic brain extraction. U-net has been a popular method for segmentation in medical images. Huang et al. applied 3D U-Net to T1-weighted structural MRI of the human brain^18^. Similarly, Pontes-Filho et al. applied a standard U-net on SE-EPI of the mouse brain, where they used a data augmentation strategy to increase the training data using elastic affine transform^30^. Hsu et al. also applied U-net to mixed T2-weighted structural MRI and GE-EPI datasets of both rats and mice, demonstrating the feasibility of processing mixed data^22^. The latter two studies used 2D U-net to process slice-by-slice instead of 3D volume. Also, these studies either used data that were acquired with a homogeneity coil or applied inhomogeneity correction as a preprocessing step. Compared to the GAN in this study, we used U-Net as the generator together with PatchGAN as the discriminator. The advantage of such a competing network design is the ability of the discriminator to learn the features of the desired output instead of relying on pixel intensity differences, which improves the generator in creating better overall results without being affected by pixel-level variations.

### Outliers

As shown in Table 1 and Fig. 4, most similarity indices of the training data are high except in certain cases. Although the predicted images of these outliers appeared to be nearly identical to their targets, we found that there were low intensity voxels after adjusting the display contrast (Supplementary Fig. 6). All corresponding training targets had the lowest intensity voxel inside the brain instead of the background. When the intensity was normalized to 0–1, the background of these training targets was not zero, as opposed to the “good” training targets, whose background value was zero. This result led to poor similarity indices, particularly for MSSIM because it is sensitive to structure, contrast and brightness differences between two images. This type of data constitutes approximately 2% of the training data.

### Limitation

This study suffers from the following limitations. First, the training and test datasets were acquired using similar coils (i.e., single-loop surface coils but different sizes for rats and mice). Because the coil intensity profile depends on the geometry of the coil in relation to the brain, images acquired by a different coil, such as an array coil, will have different intensity inhomogeneity features. The efficacy of the intensity correction under different coils remains to be evaluated. Second, the images have similar pulse sequence parameters, particularly the echo time, and resolutions. Because the echo time and spatial resolutions could affect the contrast and distortion of the EPI, they would affect the feature contents of the data and thus the performance of the trained model. Third, the “gold standard” of coil intensity correction was generated by a popular algorithm, N4. Therefore, the intensity correction by the GAN could only be as good as N4. The performance of N4 depends on the adjusted parameter, and sometimes does not generate a completely uniform profile. Better ways to generate the gold standard, such as measuring the coil B1 profile, is preferable. As the real B1 field depends on the object and EPI distortion, the B1 field acquired using a pulse sequence (e.g., conventional gradient echo) that has different distortion from the EPI sequence will result in a different B1 field. Previous studies that develop this kind of correct methods usually used synthetic data by applying an assumed bias field as weighting factor to a perfect image without bias field^31^ or used N4, as in this study^32^. Using an assumed bias field may be acceptable for structural MRI but is not suitable for highly distorted EPI. Obtaining a true “gold standard” thus remains a challenge. Fourth, brain anatomy was associated with a specific type of EPI. SE-EPI was obtained from the rat brain, and GE-EPI was obtained only from the mouse brain. There is no SE-EPI data of mouse or GE-EPI data of rat. Because the brain structures of rats and mice are similar, the influence of anatomical differences on processing performance is expected to be small. Future studies that include more diverse datasets will be required to clarify this issue. Fifth, typical multiband EPI acquisition could suffer from slice leakage artifacts that impact the image quality and bias field. The proposed multiband GE-EPI avoided the brain in different slices from overlapping with each other during acquisition and therefore did not suffer from slice leakage in typical multiband EPI on a clinical MRI scanner. However, this artifact may affect the accuracy when expanding this technique to multiband EPI data from clinical scanners. Finally, we did not have sufficient data to divide the dataset into three parts. Data augmentation is a promising technique to overcome issues associated with limited training data^33^ and to balance the number of GE-EPI and SE-EPI data in future work.

These limitations are primarily due to the fact that data were obtained from a single site. Although several recent studies have provided their image data in open repositories, they typically only provide raw images but not processed data, such as the brain mask, which are critical for training models and testing new algorithms. Future studies will benefit from open data that share the processed data. To promote this initiative, the imaging data used and analyzed in this study are available from the corresponding author on reasonable request.

## Conclusion

In this article, a deep learning model, 3D pix2pix, that is designed to combine automatic coil inhomogeneity correction and brain extraction was developed and validated using several quantized similarity indices. With sufficient training data, the model can effectively combine these two district and operator-dependent image processing steps in an advanced neuroimaging data processing pipeline. Further development and refinement of the model could allow fully automated data processing without user intervention and thus improve the efficiency of processing big data. Although this study only demonstrated the method using rodent EPI data, a similar algorithm should be trained and applied to human data.

## Methods

### Generative adversarial networks

The GAN architecture primarily has two parts, a discriminator (*D*) and a generator (*G*), which play two different roles in the algorithm. The goal of the discriminator is to distinguish whether an input image comes from the generator. Conversely, the goal of the generator is to generate a pseudoimage that can deceive the discriminator. The relationship between discriminator and generator is similar to that of a vaccine and bacteria or a predator and its prey; they have opposite purposes in the algorithm. In an iterative adversarial process, the ability of both sides increases. Therefore, an image generated by the generator will be increasingly similar to the real image. The objective function from the original paper of GANs is shown in (1):

1 $$\underset{G}{\mathrm{min}}\underset{D}{\mathrm{max}}V\left(D, G\right)= {\mathbb{E}}_{x\sim {p}_{data}\left(x\right)}\left[\mathrm{log}D\left(x\right)\right]+{\mathbb{E}}_{z\sim {p}_{z}\left(z\right)}\left[\mathrm{log}\left(1-D\left(G\left(z\right)\right)\right)\right]$$

where $x$ is data acquired from the real world, $z$ is random noise for the generator, $D\left(x\right)$ is the output of the discriminator, and $G\left(z\right)$ is the generated fake data. ${p}_{data}$ and ${p}_{z}$ are the distributions of real-world data and random variable *z*, respectively. subscript $x\sim {p}_{data}$ represents that $x$ belongs to ${p}_{data}$. ${\mathbb{E}}$ is the expected value. The designed output range of the discriminator is between 0 and 1. This objective function is expected to train the discriminator to distinguish every real-world data x from the fake data $G\left(z\right)$.

### 3D pix2pix

To process image data, we used pix2pix, which is an extended topology of GANs that can convert a picture with a certain style into another style^34^. pix2pix consists of a PatchGAN classifier as the discriminator and a U-net as the generator^35^. Different from the traditional classifier, which maps an image onto a single number, the PatchGAN classifier maps an image onto a $\mathrm{M}\times \mathrm{M}$ patch, and every element in this patch has its own receptive field. There are several benefits of the PatchGAN classifier. First, images of different matrix sizes can be verified by the same PatchGAN. Second, this method does not need to process an entire image each time so that a PatchGAN model has fewer weighting parameters and thus will be more efficient. U-net has an autoencoder-like topology. The biggest difference between U-net and autoencoder^36^ is that U-net has a shortcut between the encoder and decoder. The function of shortcut is to provide location information of a pixel of an image from the encoder block to the decoder block, making the decoder produce a higher quality image.

Particularly, pix2pix is a type of conditional GAN (cGAN)^37^ that imposes an additional condition on the discriminator and the generator. After training, the output image of the generator will be constrained by the condition, and the discriminator must distinguish the authenticity of the input image and determine whether there is a relationship between the input image and the additional condition. The condition can be a label of class, vector, a portion of data from different modalities, or even a certain type of image. The loss function of cGANs is shown in (2):

2 $${\mathcal{L}}_{cGAN}\left(G,D\right)={\mathbb{E}}_{c,x}\left[\mathrm{log}D\left(c,x\right)\right]+{\mathbb{E}}_{c}\left[\mathrm{log}\left(1-D\left(c,G\left(c\right)\right)\right)\right]$$

where $c$ is the condition. In this study, we set the raw image as $c$ and the ideally inhomogeneity corrected and brain extracted image as $x$. There are two input states for the discriminator: the first takes “c” and “x” as input, denoted as “D(c, x)”, where the variable “c” represents the raw image that is affected by intensity bias and the variable “x” represents the image with bias field corrected; and the second takes the raw image “c” and a fake image G(c) as input, denoted as D(c, G(c)). The topology of the generator only requires a single input (“c”). According to the original pix2pix paper^34^, the noise variable z is not necessary; thus, we did not use random noise as input. pix2pix then mixes the cGAN loss function with traditional loss functions, such as the L1 or L2 distance, to enhance sharpness. In this study, we used the L1 distance as in (3):

3 $${\mathcal{L}}_{L1}\left(G\right)={\mathbb{E}}_{c,x(L1)}\left[{\Vert x-G\left(c\right)\Vert }_{1}\right]$$

Therefore, the final loss function is:

4 $${G}^{*}=arg\underset{G}{\mathrm{min}}\underset{D}{\mathrm{max}}{\mathcal{L}}_{cGAN}\left(G,D\right)+\lambda {\mathcal{L}}_{L1}\left(G\right)$$

where $\lambda$ is 100, as used in the original pix2pix paper^34^. To prevent pseudoimages between slices from forming discontinuities, we expanded the original 2D framework of the pix2pix architecture into 3D so that it could properly process the volumetric data. The original pix2pix is based on a 2D framework, which means that the shape of the input/output/hidden layers of the generator/discriminator and all the calculations (e.g., convolution, padding, pooling or stride) are suitable for a two-dimensional image. In this study, we added one more dimension to the input/output/hidden layers and called the “Conv3D” and “Conv3DTranspose” functions from the Keras API to perform the calculation in three dimensions with additional parameters for the third dimension. In addition, we also modified the layer number and hyperparameters of the U-net to have 6 layers of encoders and decoders.

To find a set of hyperparameters to balance the generator and discriminator, we used a search strategy that is similar to the “grid search”. The key hyperparameters to be tuned are learning rates for the generator and discriminator. We used a list of numbers in different orders of magnitude, such as ${2\times 10}^{-3}$, ${1\times 10}^{-3}$, ${5\times 10}^{-4}$, …, ${2\times 10}^{-6}$, and ${1\times 10}^{-6}$. Based on the trends of loss in the early stage of the training process, we can determine whether to stop the process. If the process was stopped, then the next combination of learning rates was evaluated.

### Dataset

Three experiments were conducted using either spin-echo EPI (SE-EPI) of the rat brain, gradient-echo EPI (GE-EPI) of the mouse brain, or both (mix). A total of 87 rat brain scans (male Wistar rat, n = 87) and 403 mouse brain scans (male C57BL/6, 373 scans from n = 78 mice; male rTg4510 mouse, n = 30) were used in this study. Each C57BL/6 mouse was scanned with 1 or 2 sessions and in 2 to 3 repeated runs of scans acquired in each session. Only one run was acquired from each rTg4510 mouse and Wistar rat. The rat experiment was approved by the Institutional Animal Care and Use Committee of the Biomedical Sciences Institutes, A*STAR, Singapore. The mouse experiment was approved by the Animal Ethics Committee of the University of Queensland and conducted in compliance with the Queensland Animal Care and Protection Act 2001 and the current National Health and Medical Research Council Australian Code of Practice for the Care and Use of Animals for Scientific Purposes. The study was also carried out in compliance with the ARRIVE guidelines. Rat brain data were obtained from a published study^38^ and were originally acquired using a volume coil for transmission, a 15-mm single-loop coil for receiving and SE-EPI with TR/TE = 2000/45 ms, 150 or 300 repetitions, and a spatial resolution of 0.4 × 0.4 × 1 mm^3^ and 0.1 mm slice gap (for details, see^38^). Mouse brain data were acquired using a volume coil for transmission, a 10-mm loop coil for receiving and multiband GE-EPI with TR/TE = 300/15 ms, 2000 repetitions (10 min), spatial resolution of 0.3 × 0.3 × 0.5 mm^3^ and 0.1 mm slice gap (for details, see^39^). All analyses were operated in real-valued data.

The EPI time-series data were motion corrected using FSL mcflirt (https://www.fmrib.ox.ac.uk/fsl) and then averaged to obtain a mean image, which was corrected for coil inhomogeneity using N4 (implemented in ANTs; http://stnava.github.io/ANTs/) with settings that were separately adjusted for rat and mouse data. Then, automatic brain extraction was conducted using PCNN (https://sites.google.com/site/chuanglab/software/3d-pcnn), followed by manual editing. To obtain optimal results from the PCNN, the expected brain size option was particularly adjusted for each data point. The brain masks generated by the optimized PCNN without editing were used for comparison with the GAN results and resulted in an image pair composed of the motion-corrected mean image (source) and the inhomogeneity-corrected and brain-extracted image (target). These pairs were divided into three datasets (SE-EPI-only, GE-EPI-only and mixed), where the latter includes both SE-EPI and GE-EPI datasets. Each dataset was randomly split into training and test data for training and testing a different GAN (Table 3). We wanted to maximize the amount of training data; thus, we only used approximately 10% as testing data (9.8% and 9.6% for the GE-EPI and mixed data, respectively). There were 36 test samples for the GE-only model, 13 test samples for the SE-only model and 43 test samples for the mixed model. We merged the GE-only and SE-only datasets into a mixed dataset, and then split the mixed dataset into a training and testing set. The test samples were chosen randomly, and this study was performed without data augmentation.

**Table 3:** Summary of the combinations of datasets used in three experiments.

Exp	Model	Purpose	Sample size
1	GE-EPI only	Training	Scan = 367
		Test	Scan = 36
2	SE-EPI only	Training	Scan = 74
		Test	Scan = 13
3	Mix	Training	GE-EPI scan = 370 SE-EPI scan = 77 Total scan = 447
		Test	GE-EPI scan = 33 SE-EPI scan = 10 Total scan = 43

Because the input and output intensity of the network model ranged from -1 to 1, the intensity of each dataset was normalized to this range by the maximum and minimum intensities with the following equation:

5 $$\left\{ {\begin{array}{*{20}l} {\hat{A} = 2 \times \left( {\frac{A - m}{d}} \right)} \hfill \\ {m = \frac{max\left( A \right) + min\left( A \right)}{2}} \hfill \\ {d = max\left( A \right) - min\left( A \right)} \hfill \\ \end{array} } \right.$$

where $A$ is the original image and $\widehat{A}$ is the normalized image. To present the source, target and GAN outputs as positive values in the results section, their intensities were rescaled to between 0 and 1 by multiplying by 0.5 and adding 0.5.

### Implementation

**Figure 5:**
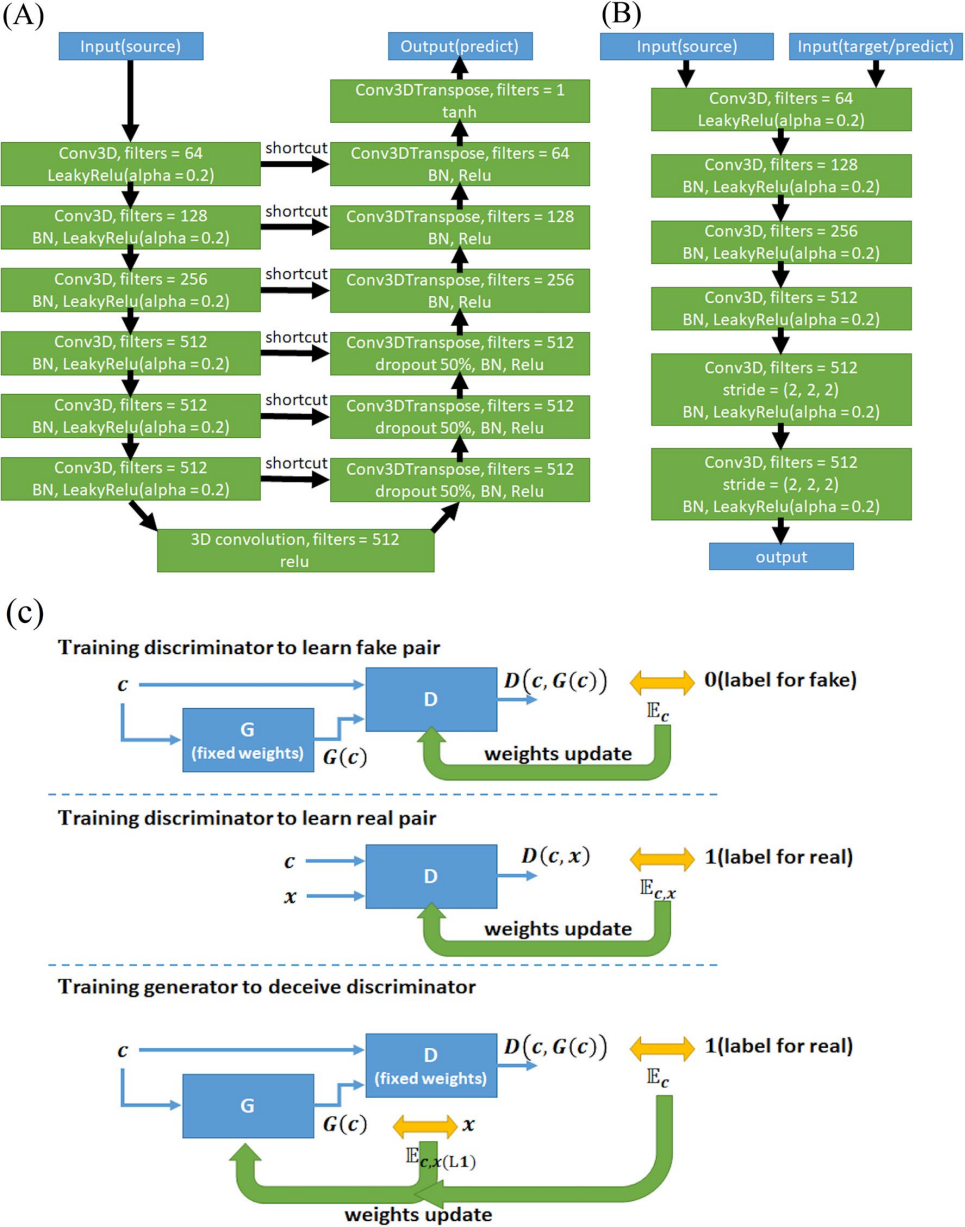
Topology of the pix2pix network. The topology and parameters of the (**A**) U-Net and (**B**) PatchGAN classifiers with two input terminals. The job of the PatchGAN classifier determines whether the two input images are the same pair and the input of the right terminal is the target (real) or prediction (fake). In the first three layers of Conv3DTranspose, dropout can prevent overfitting. “BN” is abbreviation of “BatchNormalization”. (**C**) Framework of the cGAN. The input “c” and “x” represent raw images that are affected by intensity bias and images with bias field correction, respectively.

We use an open source API, Keras (http://keras.io/), for deep-learning model building, training, prediction and testing. The program runs on a GPU-based server ESC8000 G4 with GeForce 1080 Ti (Nvidia, USA). Figure 5 shows the topology of the U-Net and PatchGAN classifiers and the parameters of U-Net, including the number of feature maps. Batch normalization was used and is denoted as BN in Fig. 5. The “shortcut” symbol represents the “concatenate” operation. The hidden layer settings are as follows: because the best initial point of the algorithm was unknown, we set the initial status of weight parameters for all layers as in the original pix2pix paper when setting the initializer, which is a normal distribution with a standard deviation of 0.02^34^. The sizes of both 3D convolution and 3D transpose convolution were 4 × 4 × 4, and both strides were 2 along each dimension. The training process included 200 epochs in total and a batch size of 1. The PatchGAN output size was 4 × 4 × 4.

Three models were trained by the 3 combinations of datasets. In each model, the weighting parameters of the model were initialized as random numbers of normal distribution. We set the epoch to 200 for model training in all experiments (mouse-only: 200 epochs × 367 samples = 73,400 iterations; rat-only: 200 × 74 = 14,800; mix: 200 × 447 = 89,400). This setting means that the training ran through up to 73,400, 14,800 and 89,400 iterations for the three datasets, respectively. The model at each epoch was saved to evaluate how the model improved over the epoch.

### Quantitative evaluation

To test the performances of the models trained by the three combinations of datasets, we use other sets of data. In particular, the rTg4510 mouse data were used to test the performance of the model that was trained solely by the C57BL6 mouse data. Both the rat and mouse testing data were applied to all three models to evaluate how well they manage images of different MRI protocols. The GAN output was compared with the reference target image and the automatic brain mask generated by one of the most popular rodent brain extraction methods, the PCNN. We used the following quantized methods to evaluate the similarity of two images: cosine angle distance (CAD)^40^, Euclidean distance (L2 norm)^40^, mean square error, peak signal-to-noise ratio, and mean structural similarity (MSSIM)^41^. Because the L2 norm, mean square error, and peak signal-to-noise ratio are linear combinations of each other, only the L2 norm is reported. In addition, the Dice index was used to compare the brain masks^42^. Their definitions are described below:

Assuming that images $A$ and *B* each have $N$ voxels, the intensity of each voxel in these images can be expressed as linear arrays (6):

6 $$A=\left[\begin{array}{l}{a}_{1}\\ {a}_{2}\\ \vdots \\ {a}_{N}\end{array}\right], B=\left[\begin{array}{c}{b}_{1}\\ {b}_{2}\\ \vdots \\ {b}_{N}\end{array}\right]$$

The following formulae were used to calculate the similarity indices.

CAD:

7 $$CAD\left(A, B\right)=\mathrm{cos}\left(\theta \right)=\frac{A\cdot B}{\Vert A\Vert \times \Vert B\Vert }$$

where CAD is the cosine angle distance between two images, and the range is [− 1, 1]. The closer the value of CAD is to 1, the more similar two images are.

L2 norm:

8 $$L2\left(A, B\right)=\sqrt{{\sum }_{i=1}^{N}{\left({a}_{i}-{b}_{i}\right)}^{2}}$$

The smallest value of the L2 norm is 0. The closer the value of the L2 norm is to 0, the more similar two images are.

MSSIM:

9 $$\left\{\begin{array}{l}SSIM\left(A, B\right)={\left[l\left(A, B\right)\right]}^{\alpha }{\left[c\left(A, B\right)\right]}^{\beta }{\left[s\left(A, B\right)\right]}^{\gamma }\\ l\left(A, B\right)=\frac{2{\mu }_{A}{\mu }_{B}+{C}_{1}}{{\left({\mu }_{A}\right)}^{2}+{\left({\mu }_{B}\right)}^{2}+{C}_{1}}\\ c\left(A, B\right)=\frac{2{\sigma }_{A}{\sigma }_{B}+{C}_{2}}{{\left({\sigma }_{A}\right)}^{2}+{\left({\sigma }_{B}\right)}^{2}+{C}_{2}}\\ s\left(A, B\right)=\frac{{\sigma }_{AB}+{C}_{3}}{{\sigma }_{A}{\sigma }_{B}+{C}_{3}}\\ {\mu }_{A}={\sum }_{i=1}^{N}{w}_{i}{a}_{i},{ \mu }_{B}={\sum }_{i=1}^{N}{w}_{i}{b}_{i}\\ {\sigma }_{A}=\sqrt{{\sum }_{i=1}^{N}{w}_{i}{\left({a}_{i}-{\mu }_{A}\right)}^{2}}, {\sigma }_{B}=\sqrt{{\sum }_{i=1}^{N}{w}_{i}{\left(b-{\mu }_{B}\right)}^{2}}\\ {\sigma }_{AB}={\sum }_{i=1}^{N}{w}_{i}\left({a}_{i}-{\mu }_{A}\right)\left({b}_{i}-{\mu }_{B}\right)\\ MSSIM\left({A}_{global},{B}_{global}\right)=\frac{1}{M}{\sum }_{i=1}^{M}SSIM\left({A}_{i}, {B}_{i}\right)\end{array}\right.$$

where ${A}_{global}$ and ${B}_{global}$ are the two images that we want to compare. $A$ and $B$ are local windows of ${A}_{global}$ and ${B}_{global}$, with ${a}_{i}$ and ${b}_{i}$ located inside windows $A$ and $B$, respectively. The SSIM is composed of three factors: luminance ($l$), contrast ($c$) and structure ($s$), which were calculated from local statistics ${\mu }_{A}$, ${\sigma }_{A}$ and ${\sigma }_{AB}$ weighted by a circular-symmetric Gaussian weighting function ${\varvec{w}}=\left\{{w}_{i}|i=1, 2,\dots ,N\right\}$ with a standard deviation of 1.5 samples, normalized to unit sum $\left({\sum }_{i=1}^{N}{w}_{i}=1\right)$. Users can decide which factor is the most important by adjusting their corresponding weights $\alpha$, $\beta$ and $\gamma$. To prevent the denominator and numerator from both being equal to zero, ${C}_{1}$, ${C}_{2}$ and ${C}_{3}$ are small scalars. In this study, $\alpha$, $\beta$, and $\gamma$ were all set to 1; and ${C}_{1}$, ${C}_{2}$, and ${C}_{3}$ were set to ${10}^{-4}$, ${9\times 10}^{-4}$, and ${4.5\times 10}^{-4}$ accordingly. $M$ is the total number of windows throughout the image, and a value of $58\times 10\times 58$ was used. The range of MSSIM is [− 1, 1], with values closer to 1 representing the highest similarity.

Dice index:

10 $$DI\left(p,\widehat{p}\right)=\frac{2\times \left|p\cap \widehat{p}\right|}{\left|p\right|+\left|\widehat{p}\right|}$$

where $p$ is the binarized target, and $\widehat{p}$ is the binarized GAN output or PCNN brain mask. An intensity threshold of 0.05 was used to turn the target and GAN output into binary masks with voxel intensity exceeding the threshold set to 1 and 0 otherwise. The range of the Dice index is between 0 and 1, with a larger value representing more overlap between two brain masks.

### Statistical analysis

To determine the performance difference between GAN models and methods, between-group comparisons were applied to the above indices using the nonparametric Friedman test (Prism, GraphPad Software, Inc., USA). P < 0.05 with correction for multiple comparisons using Dunn’s method was regarded as significant. Except particularly noted, values are reported as the mean ± standard deviation.

## Supplementary Information

Supplementary Legends.

Supplementary Figure 1.

Supplementary Figure 2.

Supplementary Figure 3.

Supplementary Figure 4.

Supplementary Figure 5.

Supplementary Figure 6.

## Data Availability

The datasets used and analyzed during the current study are available from the corresponding author on reasonable request.
