# Supplementary material for: Deep learning network for integrated coil inhomogeneity correction and brain extraction of mixed MRI data
Source: Sci Rep. 2022 May 20;12:8578. doi: 10.1038/s41598-022-12587-6 (PMC9123199; doi:10.1038/s41598-022-12587-6)
Supplement: Supplementary file 1 — Supplementary Legends. [file 41598_2022_12587_MOESM1_ESM.docx]

**Supplementary data**

Supp Fig. 1. Example outputs of the GAN trained by the GE-EPI-only dataset. The left column shows the results of the model applied to the training data, where (A) is the training source and (B) is the target. (C-G) are the outputs at 5 selected iterations with the image in (A) as the input. The right column shows the results of test data, where (H) is the source and (I) is the target. (J-N) are the outputs from the five iterations using (H) as the input. For each column, the left frame is the histogram of the image, and the right is the image itself. The histogram shows the intensity range from 0.05 to 1 divided into 200 bins on a log scale. (B) and (I) are reference images, and (G) and (N) are the “fake” images generated by the GAN.

Supp Fig. 2. Example outputs of the GAN trained by the SE-EPI-only dataset. The left column shows the results of training data, where (A) is the training source and (B) is the target. (C-G) are the outputs at 5 selected iterations with the image in (A) as the input. The right column shows the results of the model applied to the test data, where (H) is the source and (I) is the target. (J-N) are the outputs from the five iterations using (H) as the input. For each column, the left frame is the histogram of the image, and the right is the image itself. The histogram shows the intensity range from 0.05 to 1 divided into 200 bins on a log scale. (B) and (I) are reference images, and (G) and (N) are the “fake” images generated by the GAN.

Supp Fig. 3. GE-EPI example outputs of the GAN trained by the mixed dataset. The left column shows the results of the model applied to the training data, where (A) is the training source and (B) is the target. (C-G) are the outputs at 5 selected iterations with the image in (A) as the input. The right column shows the results of the model applied to the test data, where (H) is the source and (I) is the target. (J-N) are the outputs from the five iterations using (H) as the input. For each column, the left frame is the histogram of the image, and the right is the image itself. The histogram shows the intensity range from 0.05 to 1 divided into 200 bins on a log scale. (B) and (I) are reference images, and (G) and (N) are the “fake” images generated by the GAN.

Supp Fig. 4. SE-EPI example outputs of the GAN trained by the mixed dataset. The left column shows the results of the model applied to the training data, where (A) is the training source and (B) is the target. (C-G) are the outputs at 5 selected iterations with the image in (A) as the input. The right column shows the results of the model applied to the test data, where (H) is the source and (I) is the target. (J-N) are the outputs from the five iterations using (H) as the input. For each column, the left frame is the histogram of the image, and the right is the image itself. The histogram shows the intensity range from 0.05 to 1 divided into 200 bins on a log scale. (B) and (I) are reference images, and (G) and (N) are the “fake” images generated by the GAN.

Supp Fig. 5. Similarity indices of training data over iterations. The CAD (first column), L2 norm (second column) and MSSIM (third column) of the GE-EPI-only model (Exp 1; A-C), SE-EPI-only model (Exp 2; D-F), and mixed-trained model (Exp 3; G-I). We calculated the mean and standard deviation of the similarity indices of all the training data in each experiment. The unit of the x-axis is the number of iterations.

Supp Fig. 6. Example of outliers in the training dataset. (A) GE-EPI data with a low MSSIM of 0.379 between target and GAN prediction. In the contrast-amplified image (left column), particularly dark areas are shown in the source and target images, but fewer are shown in the GAN prediction, leading to a mismatch. From top to bottom, the right column shows the original source, target and prediction. (B) Example of typical GE-EPI with a very high MSSIM of 0.994. There is no dark area seen in the image.
