# Supplementary figures and images for: Deep learning network for integrated coil inhomogeneity correction and brain extraction of mixed MRI data
Source: Sci Rep. 2022 May 20;12:8578. doi: 10.1038/s41598-022-12587-6 (PMC9123199; doi:10.1038/s41598-022-12587-6)

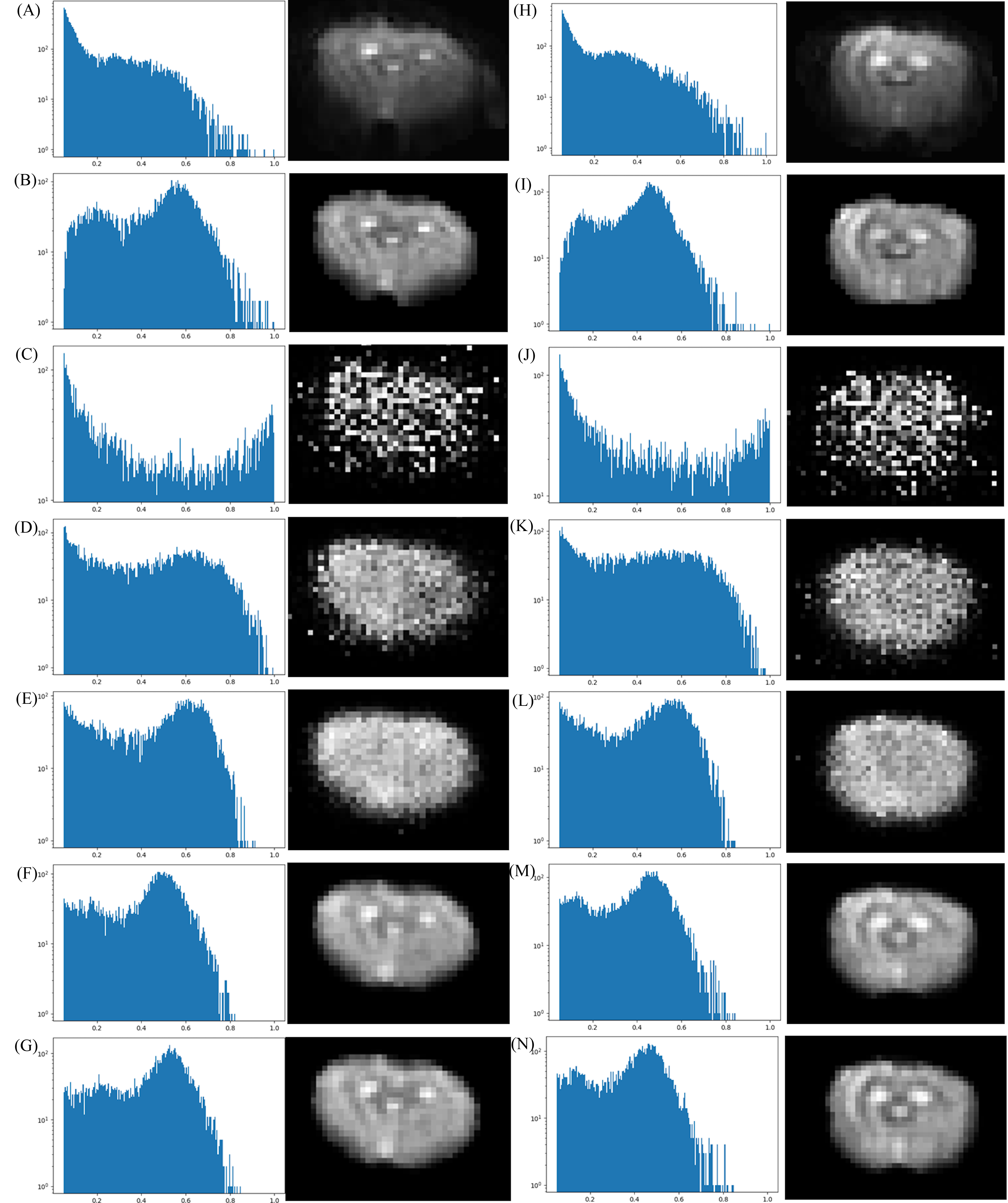

Supplement: Supplementary file 2 — Supplementary Figure 1. [file 41598_2022_12587_MOESM2_ESM.tif]

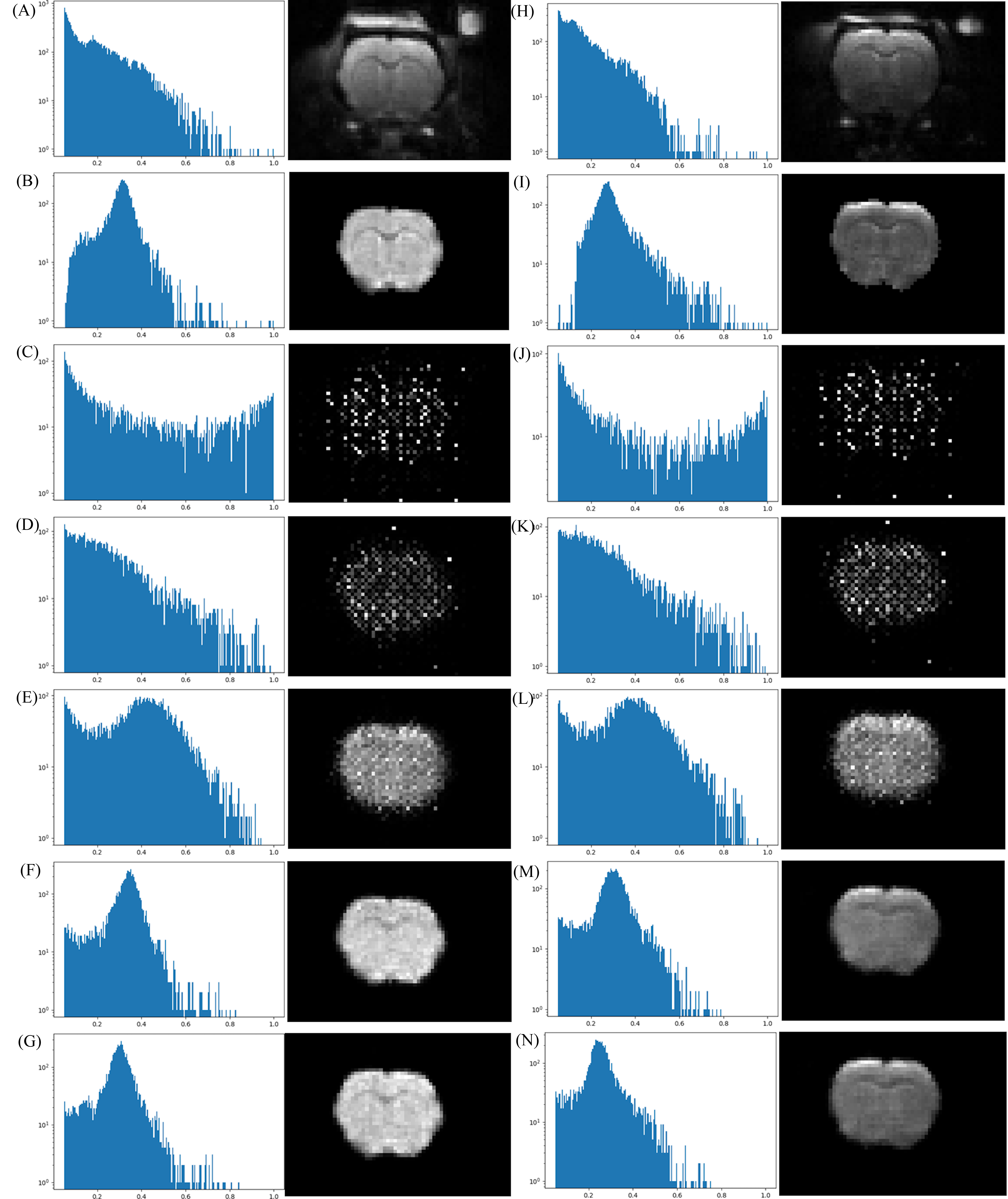

Supplement: Supplementary file 3 — Supplementary Figure 2. [file 41598_2022_12587_MOESM3_ESM.tif]

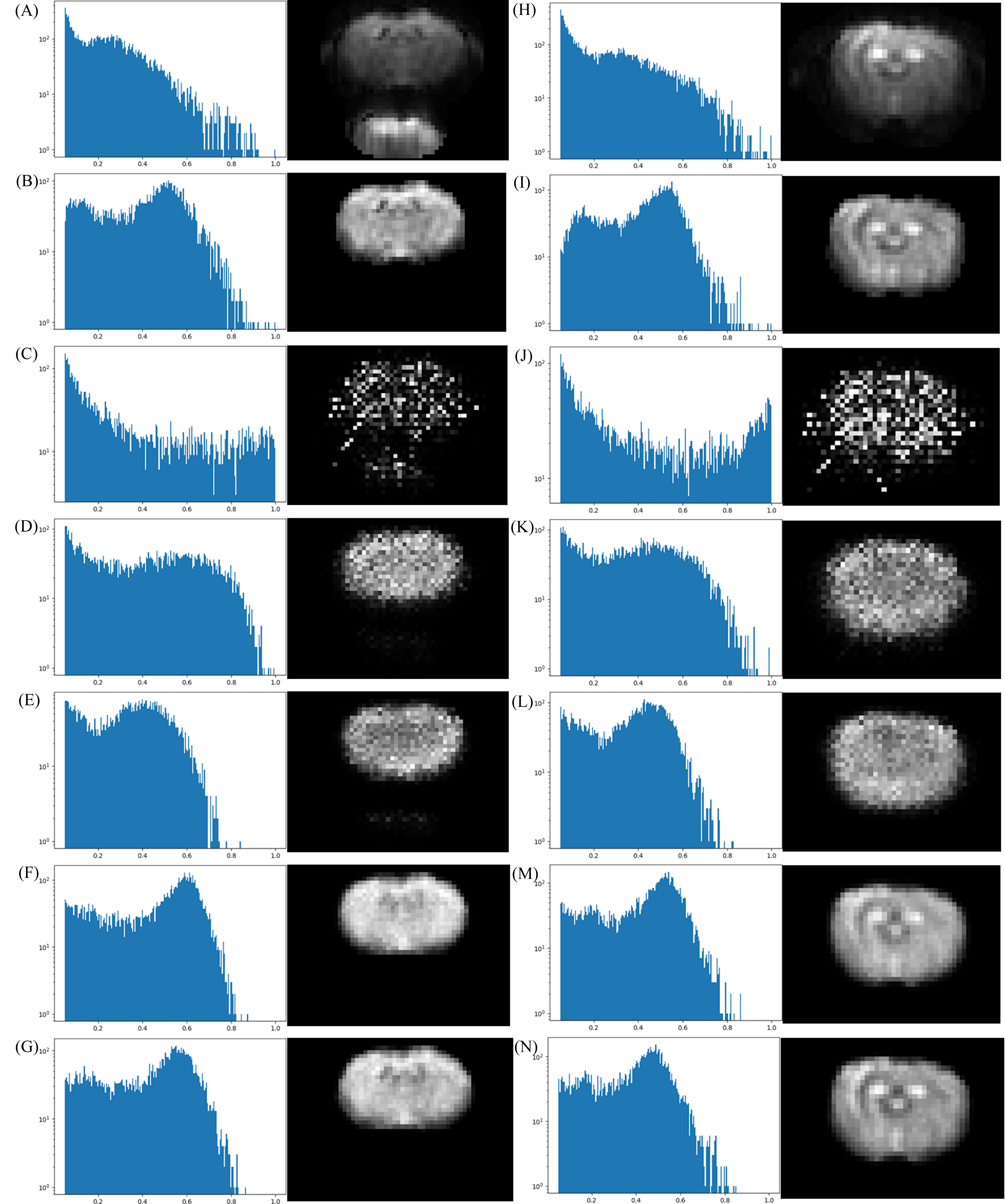

Supplement: Supplementary file 4 — Supplementary Figure 3. [file 41598_2022_12587_MOESM4_ESM.tif]

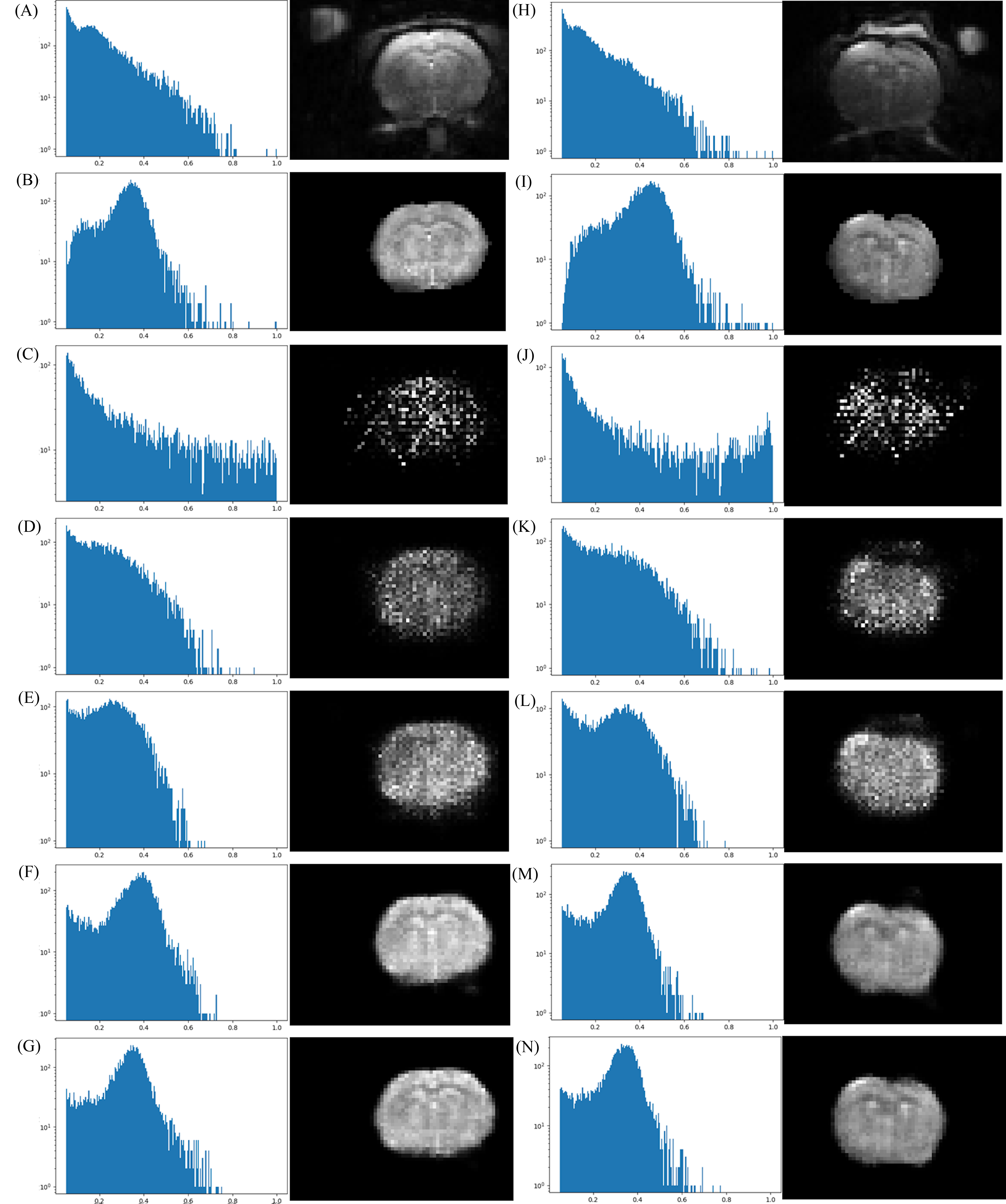

Supplement: Supplementary file 5 — Supplementary Figure 4. [file 41598_2022_12587_MOESM5_ESM.tif]

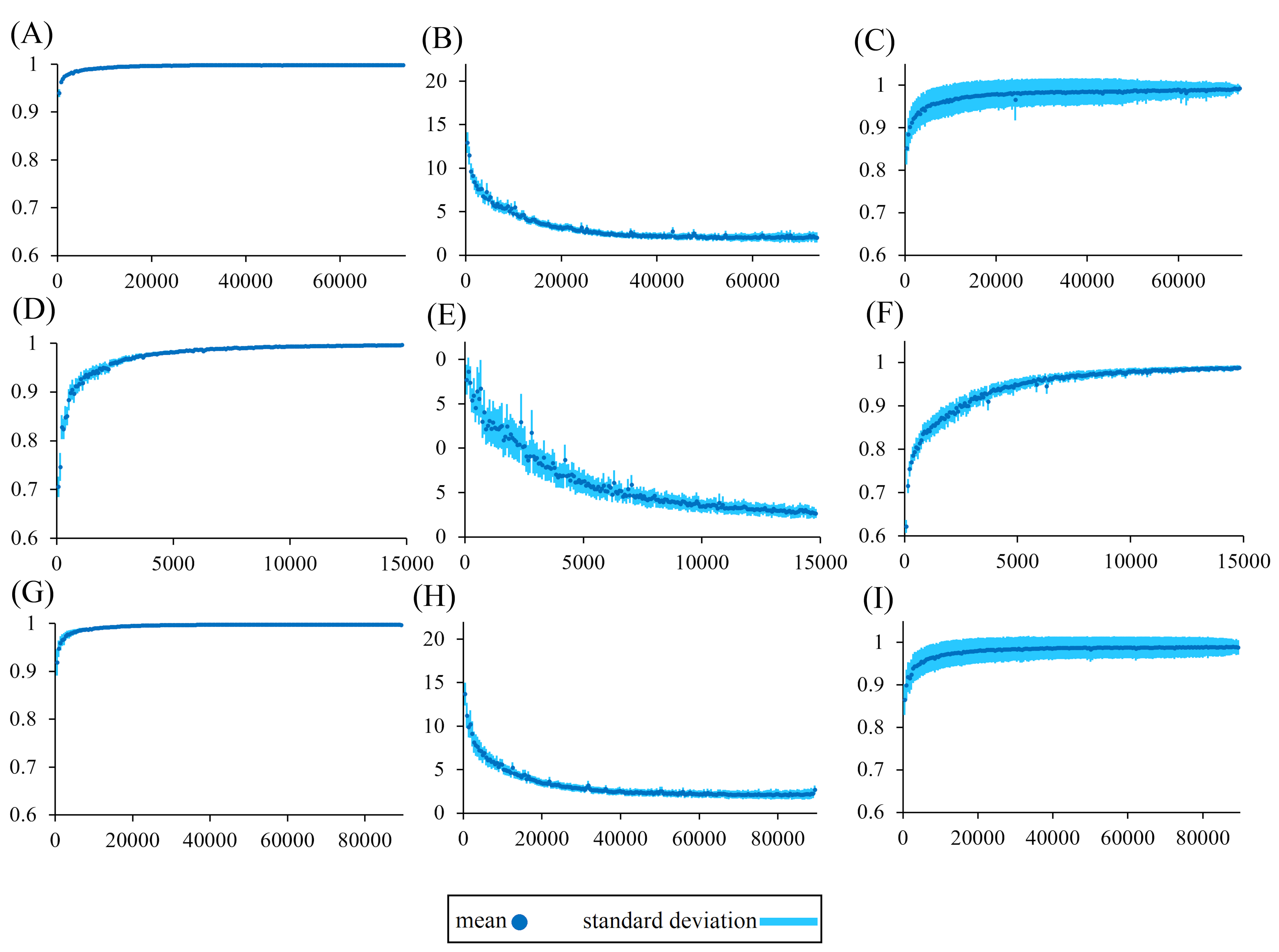

Supplement: Supplementary file 6 — Supplementary Figure 5. [file 41598_2022_12587_MOESM6_ESM.tif]

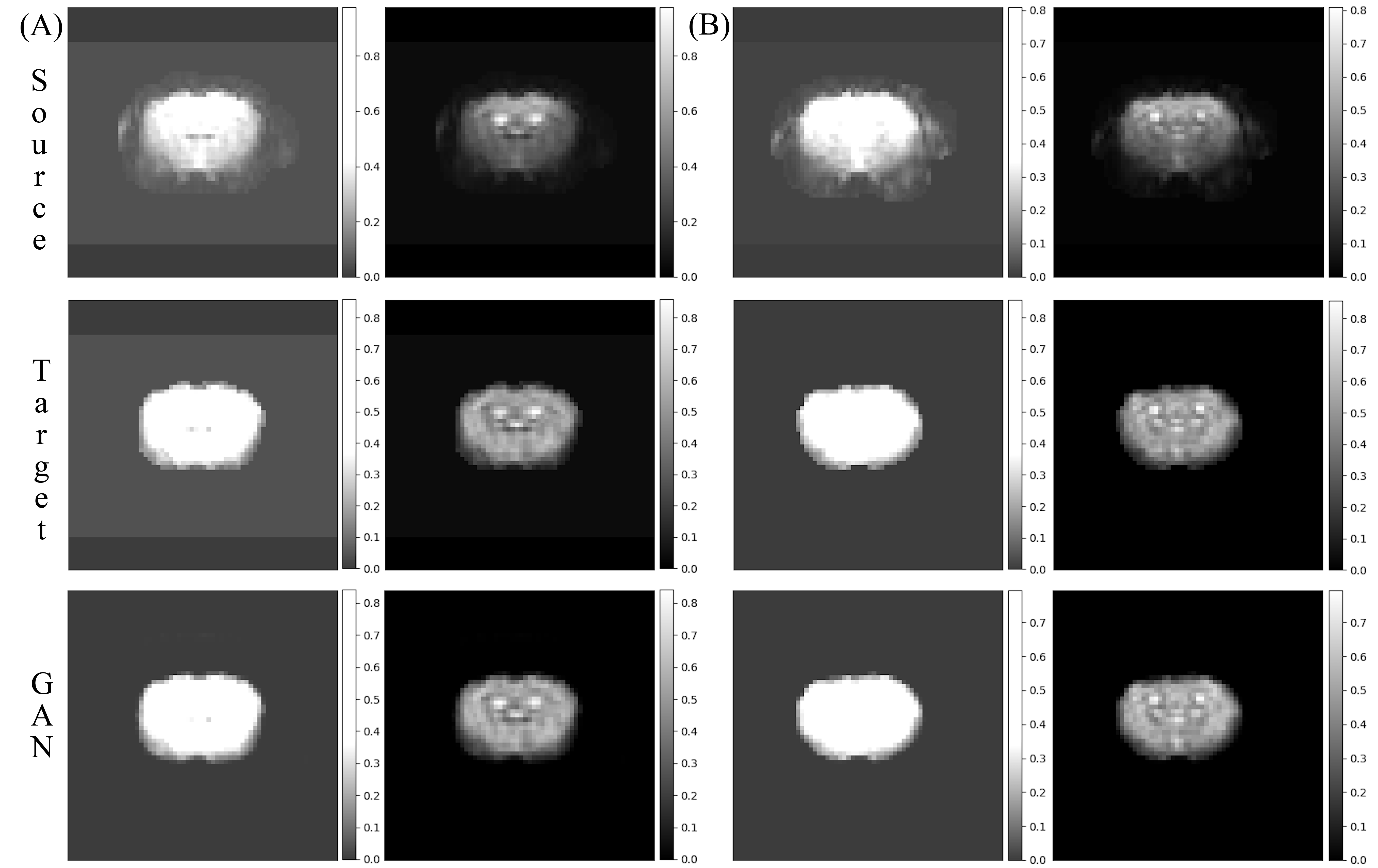

Supplement: Supplementary file 7 — Supplementary Figure 6. [file 41598_2022_12587_MOESM7_ESM.tif]
